# Biliary tree neuroendocrine tumor, an incidental finding

**DOI:** 10.1016/j.ijscr.2021.105940

**Published:** 2021-04-30

**Authors:** Monther Altiti, Ahmad Jabr Al-sa'afin, Tayseer Al-tawarah, Ahmad Suleihat, Saleh Abulhaj, Mohamad Mahseeri

**Affiliations:** a General Surgery Department, School of Medicine, University of Jordan, Jordan; b Emergency Medicine Department, School of Medicine, University of Jordan, Jordan; c General Surgery Department, School of Medicine, Muta University, Jordan

**Keywords:** Neuroendocrine, Biliary tree, Jaundice, Carcinoid

## Abstract

**Introduction and importance:**

Biliary tree neuroendocrine tumors (NET) are rare, with 100 cases in the literature, and have an excellent prognosis. Although they are rarely diagnosed before surgery, complete surgical excision offers optimal treatment.

**Case presentation:**

We report a case of a 36-year-old female patient referred to the surgical team with obstructive jaundice for which she was investigated and found to have a common bile duct tumor showing proximal obstruction. Excision of the tumor with hepaticojejunostomy was done. Later on, the pathology report showed grade-1, well-differentiated neuroendocrine carcinoma, which was completely excised. No further intervention was provided to the patient.

**Clinical discussion:**

Complete surgical resection with excision of the extrahepatic bile ducts and portal lymphadenectomy in addition to Roux-en-Y hepaticojejunostomy or even pancreaticoduodenectomy for distal CBD neuroendocrine tumors gives sufficient treatment in the majority of cases. No evidence of the advantage of chemo-radiotherapy as part of the treatment for this tumor.

**Conclusion:**

Biliary tree neuroendocrine tumors are benign tumors, and it is usually difficult to ascertain the diagnosis preoperatively. However, complete surgical excision offers an optimal treatment with no evidence of chemotherapy or radiotherapy's role in the management.

## Highlights

• Biliary tree NETs have an excellent prognosis with a 10-year survival of 80%
• complete surgical excision offers optimal treatment for biliary tree NET
• Size of the tumor, presence of lymphovascular invasion, and Ki-67 assesses prognosis

## 1 Introduction

Neuroendocrine tumors (NET) arise from enterochromaffin cells of the gastrointestinal and pancreatic tract. Commonly referred to as carcinoid tumors and usually present in the small intestine and rarely found within the bile ducts [1]. This low incidence could be explained by the minor presence of Kulchitsky cells in the extrahepatic biliary tree, which predisposes to the disease. While At least 95% of all neoplasms in the extrahepatic bile duct are adenocarcinomas, only 0.2–0.3% of neuroendocrine tumors arise from this site [2].

Preoperative diagnosis of biliary tree carcinoid is rare; most of the cases presented in the literature were either diagnosed incidentally intra-operatively or found on the histopathology report postoperatively. Further, no evidence of the advantage of chemo-radiotherapy as part of the treatment for this tumor.

Here we present a case of common bile duct NET treated with hepaticojejunostomy and found to be a NET postoperatively on the pathology report. This work has been reported in line with the PROCESS criteria [3].

## 2 Case presentation

A 36-year-old housewife, not known to have any previous chronic medical illnesses or family history of gastrointestinal malignancy, referred to our team with generalized itching that started one 1-month earlier; she also had noticed a change in urine and stool color with yellowish discoloration of her sclera. She did not document any history of abdominal pain nor weight loss. She was not alcoholic, and No previous history of surgery nor endoscopic retrograde cholangiopancreatography (ERCP) was documented. Her abdominal examination showed no palpable masses, and her liver could not be palpated with jaundice being evident.

Her labs showed increased bilirubin level and evidence of obstructive jaundice, with other liver enzymes being elevated. Her CBC, INR, amylase, albumin, ESR, CRP, AMA, ANA, and tumor markers were within normal range. An initial ultrasound showed intrahepatic biliary tree dilatation with no evidence of gallbladder stones.

**Fig. 1:**
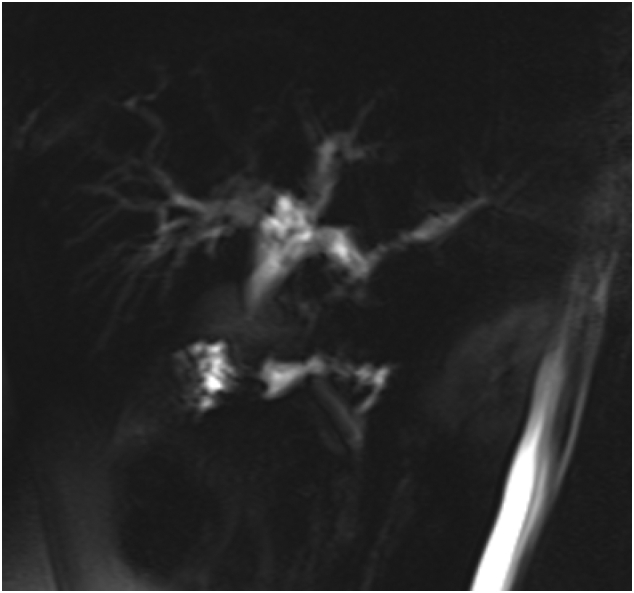
Rounded porta hepatis soft tissue lesion measuring compression of the common hepatic duct with proximal intrahepatic biliary tree dilatation.

**Fig. 2:**
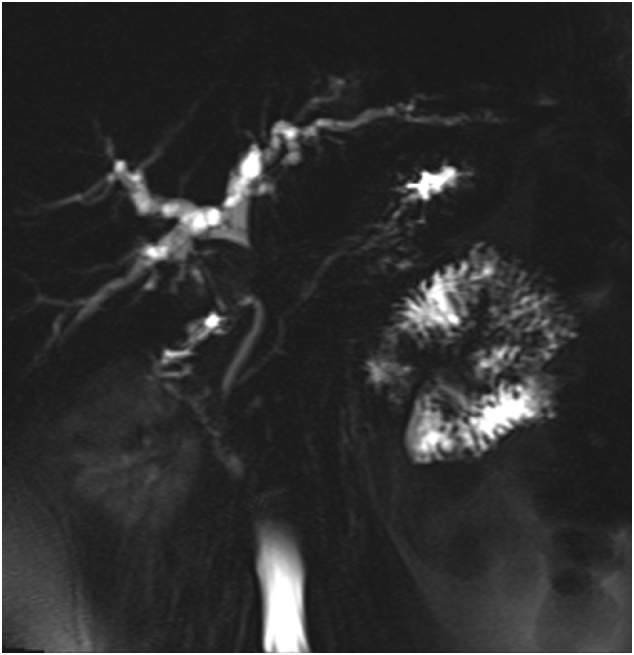
A stricture at the proximal common bile duct with proximal intrahepatic biliary tree dilatation.

Magnetic resonance cholangiopancreatography (MRCP) [Fig. 1, Fig. 2] showed rounded porta hepatis soft tissue lesion measuring 2 ∗ 2.4 ∗ 2.8 cm, causing marked compression on common hepatic duct with proximal intrahepatic biliary tree dilatation and indenting the gallbladder fundus and Cystic duct.

**Fig. 3:**
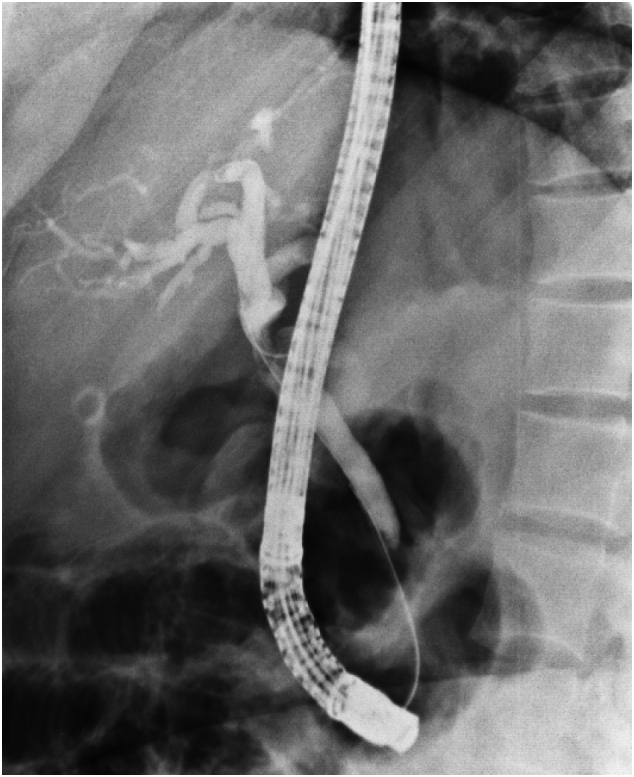
A stricture just distal to the common hepatic duct confluence and proximal intrahepatic biliary tree dilatation.

Computed tomography (CT) scan with contrast of chest and abdomen and pelvic showed enhancing soft tissue lesion in the porta hepatis with no evidence of distant metastasis. ERCP [Fig. 3] was done, showing a common hepatic duct stricture extending to just below the hepatic confluence. Brush cytology was done; however, no specific pathology has been identified, and a plastic stent was inserted for biliary decompression.

After the diagnosis with obstructive jaundice and the evidence of stricture in the common bile duct caused by a soft tissue lesion in the porta hepatis, biliary adenocarcinoma, and biliary stricture was the most likely differential diagnosis. The plan was discussed with the patient, and the decision was planned for surgery. Intra-operatively, a stricture along the proximal common bile duct was seen, for which the patient underwent Roux en-Y hepaticojejunostomy with excision of the tumor along with common bile duct (CBD) and gallbladder. A hepatobiliary consultant with a 20-year of experience conducted the procedure.

Her postoperative course was uneventful, with improving liver function values. She was discharged home on the fifth day postoperatively. At her two weeks' follow-up, she had complete resolution of her symptoms and a standard liver function test.

**Fig. 4:**
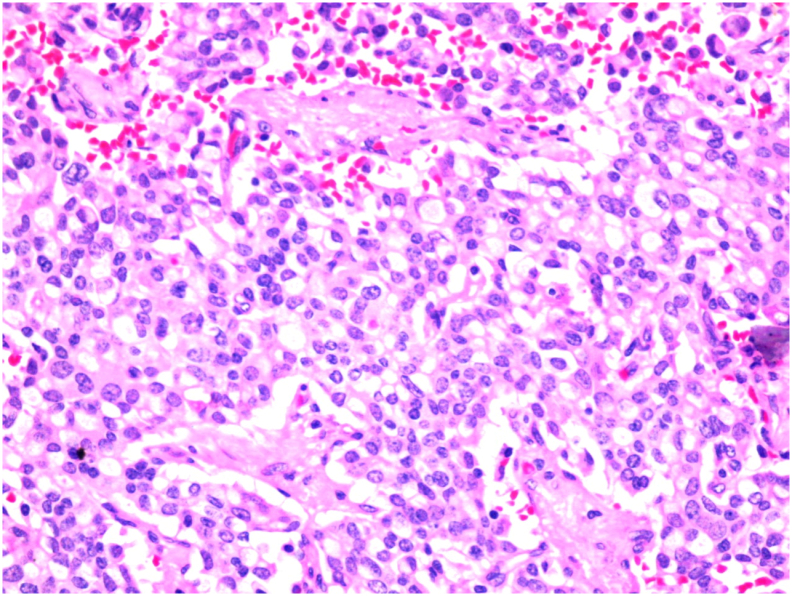
A proliferation of pleomorphic round to oval cells with a moderate amount of finely granular cytoplasm.

**Fig. 5:**
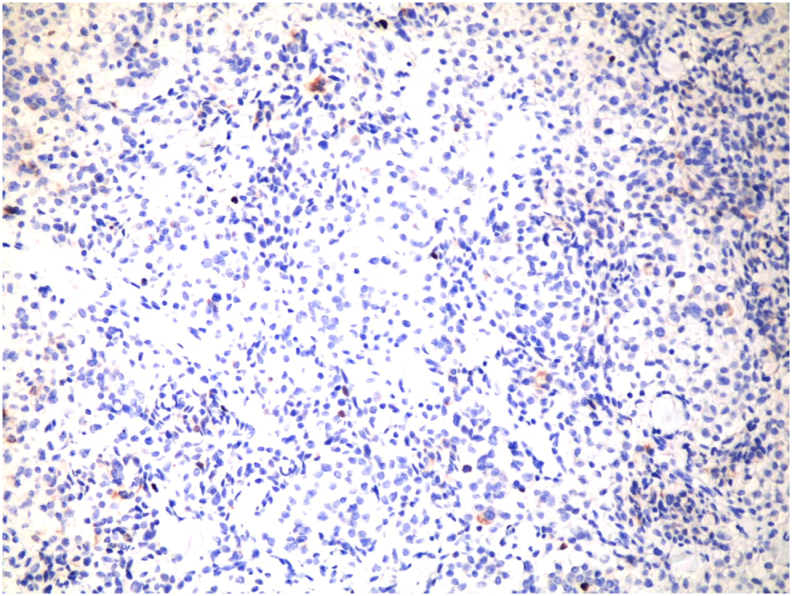
Salt and pepper appearance of the nuclei.

The pathology [Fig. 4, Fig. 5] showed a proliferation of pleomorphic round to oval cells with a moderate amount of finely granular cytoplasm. The nuclei show a salt and pepper appearance. The mitotic index is less than 1mitosis/10HPF. The tumor cells are invading the mucosa of the common bile duct and the surrounding fat. Perineural invasion and lymphovascular invasion are seen. Chromogranin and Synaptophysin immunohistochemical stains were done, and they were positive in the appearing neuroendocrine cells and the signet appearing cells. Ki67 immunohistochemical stain was positive in less than 2% of the tumor suggestive of well-differentiated neuroendocrine carcinoma, grade 1.

Later follow-up after a one-year interval did not show metastasis, and the patient was doing fine without any complications.

## 3 Discussion

Neuroendocrine tumors constitute a specific histological pattern of tumors, and they were first described by Lubarsch in 1888. To this date, its origin is still unclear. It has been suggested that inflammation causes metaplasia of Kulchitsky's cells, therefore causing their malignant potential. [4]

Common bile duct [CBD] tumors represent less than 2% of all cancers, usually cholangiocarcinomas [80%]. Neuroendocrine tumors of the bile ducts account only for 0.2% to 2.0% of all gastrointestinal NET [4]. CBD is the most common location for extrahepatic carcinoid tumors, followed by a perihilar duct, cystic duct, and common hepatic duct [4,6]. Gallstones and choledochal cyst von Hippel-Lindau syndrome, multiple endocrine neoplasia [MEN-I] syndrome, or the Zollinger-Ellison syndrome have been investigated as a risk factor for the disease [7].

Our patient had symptoms suggestive of obstructive jaundice, resembling most of the literature. Obstructive jaundice, biliary colic, and pruritus represent the most documented symptoms. Patients with obstructive jaundice are investigated with imaging and brush cytology, and no specific pathology could be found. This is further augmented in neuroendocrine tumors because of the submucosal location of the pathology, which yields a high false-negative result of the cytology [5].

To this date, 150 cases of extrahepatic biliary NET have been reported. Since the first case described in 1959, only 4 cases had a preoperative diagnosis of carcinoid tumors [5.1%], with most cases being diagnosed intra-operatively or even postoperatively on the histopathology report like our case [1]. This is due to the absence of hormonal and serum markers on preoperative investigations, in addition to the submucosal location of the tumors as described earlier. However, some authors reported chromogranin, somatostatin, serotonin, and gastrin to be high in these patients [2].

Several reports described some characteristics to differentiate cholangiocarcinoma from NET based on history [8]. Other reports suggested a differentiation based on imaging. NET has a well-circumscribed wall enhancing on arterial phase on CT scan with a polyp-like appearance of a small size, which may distinguish it from cholangiocarcinoma, which usually has a mass obstructing the lumen with hepatic metastasis evident on the liver [6,9].

CT scan, MRCP, and ERCP are the investigation of choice for obstructive jaundice cases. NETs have a specific feature of somatostatin receptors. As a result, DOTA-TATE, DOTATOC, and DOTANOC scans can visualize the tumor. Unfortunately, this is only valid in metastatic disease [7]. Moreover, Positron emission tomography (*PET*)/CT has no benefit in detecting these tumors [6]. Since our patient had a low-grade, well-differentiated tumor, a DOTA scan was not done.

Complete surgical resection with excision of the extrahepatic bile ducts and portal lymphadenectomy in addition to Roux-en-Y hepaticojejunostomy or even pancreaticoduodenectomy for distal CBD neuroendocrine tumors gives sufficient treatment in the majority of cases [4,7].

The role of adjuvant chemotherapy is still questionable about NETs, as most authors could not describe any survival benefits [4]. In the minority of cases, when a proven preoperative diagnosis of NET is found, Hazama et al. suggested that if there is a biopsy-proven preoperative diagnosis of NEC, preoperative chemotherapy will improve prognosis compared to surgery alone or surgery with adjuvant chemotherapy [5].

Size of the tumor, presence of lymphovascular invasion, and the quantitative assessment of Ki-67 reactive cells, Mitosis index, together with tumor differentiation, and grade help to determine prognosis, although no absolute criteria for determining the malignant potential of extrahepatic NETs have been published to this date; generally, they have a 10-year survival of 80% [7]. In his report of 47 patients, Lee WJ et al. demonstrated that patients with ampulla of Vater NET have a better prognosis and earlier diagnosis than biliary tree NET [10].

Only 40% of patients with NET have hepatic metastases at diagnosis, with the liver being the most affected site [11]. Safwan M et al. reported that serum levels of CgA correlate significantly with metastatic disease, and it is beneficial for recording the recurrence of the disease [8]. In such circumstances, chemoembolization and alcohol-based embolization, together with high-frequency ablation, can be utilized with a successful outcome [12].

This study has multiple limitations; they include a short period of follow-up with clinic visits for one year only, and a DOTA scan was not done because the histopathology showed a low-grade, well-differentiated tumor, and it might have a role in the evaluation of metastasis.

## 4 Conclusion

Biliary tree neuroendocrine tumors are benign tumors, and it is usually difficult to ascertain the diagnosis preoperatively. However, complete surgical excision offers an optimal treatment with no evidence of chemotherapy or radiotherapy's role in the management.

## 5 Learning points

• Neuroendocrine tumors usually present with obstructive jaundice and can be initially misdiagnosed as cholangiocarcinoma.
• Biliary tree NETs are rare and have an excellent prognosis with a 10-year survival of 80%.
• Although they are rarely diagnosed before surgery, complete surgical excision offers an optimal treatment with no evidence of chemotherapy or radiotherapy's role in the management.
• Prognosis is assessed by the Size of the tumor, lymphovascular invasion, and Ki-67 on reactive cells.

## Consent

Written informed consent was obtained from the patient for publication of this case report and accompanying images. A copy of the written consent is available for review by the Editor-in-Chief of this journal on request.

## Provenance and peer review

Not commissioned, externally peer-reviewed.

## Sources of funding

No funding.

## Ethical approval

This article is approved by the ethics committee of Jordan University Hospital and IRP of the University of Jordan.

## Author contribution

Mohamad Mahseer MD.: writing and editing and reviewing.

Monther Altiti MD: reviewing and editing.

Ahmad Jabr Al-sa'afin MD: reviewing.

Tayseer Al-tawarah MD.: writing and editing.

Ahmad Suleihat MD: reviewing.

Saleh Abulhaj MBBS.: writing and editing.

Research registration: N\A.

Guarantor: Mohamad Mahseeri MD. RCSEd.

## Declaration of competing interest

No conflicts of interest.
